# Dengue Surveillance in Veterans Affairs Healthcare Facilities, 2007–2010

**DOI:** 10.1371/journal.pntd.0002040

**Published:** 2013-03-14

**Authors:** Patricia L. Schirmer, Cynthia A. Lucero-Obusan, Stephen R. Benoit, Luis M. Santiago, Danielle Stanek, Achintya Dey, Mirsonia Martinez, Gina Oda, Mark Holodniy

**Affiliations:** 1 Office of Public Health, Department of Veterans Affairs, Palo Alto, California, United States of America; 2 Public Health Surveillance Program Office, Office of Surveillance, Epidemiology, and Laboratory Services, Centers for Disease Control and Prevention, Atlanta, Georgia, United States of America; 3 Dengue Branch, Division of Vector-Borne Diseases, Centers for Disease Control and Prevention, San Juan, Puerto Rico, United States of America; 4 Bureau of Environmental Public Health Medicine, Florida Department of Health, Tallahassee, Florida, United States of America; 5 Veterans Affairs Caribbean Healthcare System - San Juan, Department of Veterans Affairs, San Juan, Puerto Rico, United States of America; 6 Division of Infectious Diseases and Geographic Medicine, Stanford University, Palo Alto, California, United States of America; University of California, Davis, United States of America

## Abstract

**Background:**

Although dengue is endemic in Puerto Rico (PR), 2007 and 2010 were recognized as epidemic years. In the continental United States (US), outside of the Texas-Mexico border, there had not been a dengue outbreak since 1946 until dengue re-emerged in Key West, Florida (FL), in 2009–2010. The objective of this study was to use electronic and manual surveillance systems to identify dengue cases in Veterans Affairs (VA) healthcare facilities and then to clinically compare dengue cases in Veterans presenting for care in PR and in FL.

**Methodology:**

Outpatient encounters from 1/2007–12/2010 and inpatient admissions (only available from 10/2009–12/2010) with dengue diagnostic codes at all VA facilities were identified using VA's Electronic Surveillance System for Early Notification of Community-based Epidemics (ESSENCE). Additional case sources included VA data from Centers for Disease Control and Prevention BioSense and VA infection preventionists. Case reviews were performed. Categorical data was compared using Mantel-Haenszel or Fisher Exact tests and continuous variables using t-tests. Dengue case residence was mapped.

**Findings:**

Two hundred eighty-eight and 21 PR and FL dengue cases respectively were identified. Of 21 FL cases, 12 were exposed in Key West and 9 were imported. During epidemic years, FL cases had significantly increased dengue testing and intensive care admissions, but lower hospitalization rates and headache or eye pain symptoms compared to PR cases. There were no significant differences in clinical symptoms, laboratory abnormalities or outcomes between epidemic and non-epidemic year cases in FL and PR. Confirmed/probable cases were significantly more likely to be hospitalized and have thrombocytopenia or leukopenia compared to suspected cases.

**Conclusions:**

Dengue re-introduction in the continental US warrants increased dengue surveillance and education in VA. Throughout VA, under-testing of suspected cases highlights the need to emphasize use of diagnostic testing to better understand the magnitude of dengue among Veterans.

## Author Summary

Dengue is an important tropical disease seen throughout the world in tropical climate zones and is spread by *Aedes* mosquitoes. Most cases of dengue in the continental US are imported. In July 2009 through 2010, dengue virus was found to be circulating in Key West, Florida (FL). Dengue virus has been transmitted in Puerto Rico (PR) for many years. This study used electronic and manual surveillance systems to identify dengue cases in VA healthcare facilities and clinically compared dengue cases in Veterans presenting for care in PR as well as in FL. We found that FL dengue cases were similar to those in PR and that Centers for Disease Control and Prevention defined confirmed/probable cases were more likely to be hospitalized within our VA system, and have either lower platelet or white blood cell counts than suspected cases. During July 2009–2010, FL cases were more likely to be tested for dengue and have intensive care admissions, but had lower hospitalization rates and headache or eye pain symptoms compared to PR cases. No one method of capturing dengue cases was perfect. It is important to educate healthcare workers about this disease to help with direct patient care as well as surveillance.

## Introduction

Dengue virus (DENV), a flavivirus with 4 serotypes, transmitted by *Aedes* mosquitoes can cause a spectrum of disease from a mild febrile illness with constitutional symptoms to a severe hemorrhagic illness [1], [2]. An important risk factor for severe dengue, dengue hemorrhagic fever (DHF) or dengue shock syndrome (DSS), is a previous infection with another DENV serotype [2]. Dengue is seen throughout the world in tropical regions and has been endemic in Puerto Rico for many years [3], [4], [5]. More recently, 2007 and 2010 were recognized as epidemic years in Puerto Rico with increased rates of dengue cases reported [3], [4], [5]. In the continental United States (US), outside of the Texas-Mexico border, there had not been a dengue outbreak since 1946 until 2009–2010 when there was an outbreak of locally acquired dengue (DENV-1) in Key West, Florida [6], [7], [8], [9], [10]. Why DENV circulated in Key West, Florida, starting in July 2009 through 2010 is not entirely clear.

In Florida and Puerto Rico, dengue was a reportable disease to the department of health prior to the identification of endemic cases in Florida in 2010. However, because of the rise in the number of dengue cases and the risk of transmission in the continental US, in 2010, dengue became one of Centers for Disease Control and Prevention's (CDC) nationally notifiable diseases based on the Council of State and Territorial Epidemiologists (CSTE) dengue case definition [11]. The Department of Veterans Affairs (VA) medical facilities, particularly in Puerto Rico and Florida, have started to perform surveillance for and report new cases of dengue to county and state/territory health departments.

The objective of this study was to evaluate dengue cases identified in VA facilities by electronic and manual methods. We subsequently compared clinical symptoms, laboratory data, illness severity, and differences between confirmed/probable and suspected cases presenting for care in Puerto Rico from 2007–2010 (including the epidemic years of 2007 and 2010) and compared these cases to dengue cases identified in Florida VA facilities between 2007 and 2010 (including the epidemic years of 2009 and 2010) to better characterize dengue identified in Puerto Rico and Florida VA facilities.

## Methods

### Ethics Statement

This project was approved by the Stanford University Institutional Review Board. The Human Subjects Research Panel at Stanford University determined that the study entitled “Healthcare-Associated Infections and Syndromic Surveillance in the Department of Veterans Affairs” met the requirements of regulation OHRP 45 CFR 46.116 (d): Requests for waiver or alteration of the informed consent process, in research that is not subject to FDA regulation in that: (1) The research involved no more than minimal risk to the subjects; (2) the waiver or alteration would not adversely affect the rights and welfare of the subjects; (3) the research could not practicably be carried out without the waiver or alteration and (4) the subjects would be provided with additional pertinent information after participation. This study was approved because the data used for its conduct was retrospective and would be obtained through subject electronic medical records (EMR) from primary care doctors, thus it was not anticipated that any situation would arise in which pertinent information would need to be shared with individual subjects. All data extracted from the medical record during the public health investigation was analyzed anonymously. We publish findings from this study and established the database regarding infection control as a tool for providers, thus patients would learn and benefit from this study through the care provided by their primary care doctors.

Dengue surveillance in the VA was done by a combination of electronic and manual surveillance. VA's Electronic Surveillance System for the Early Notification of Community-based Epidemics (ESSENCE) biosurveillance system was used to identify outpatient and emergency department visits (January 2007–December 2010) and inpatient admissions (available data from October 2009–December 2010) with dengue ICD-9 codes (061 and 065.4) at all 152 VA medical centers and over 970 outpatient clinics in the United States and its territories, including 5 facilities in Puerto Rico and 67 in Florida [12], [13]. Cases were also identified in Florida using VA data transmitted to CDC's BioSense system for 2010. BioSense captured possible dengue cases using dengue ICD-9 codes as well as syndromic definitions (fever with rash; fever with unexplained bleeding; or fever with thrombocytopenia) [14]. We also identified cases that were manually collected by infection preventionists in Florida and Puerto Rico and reported to VA's Office of Public Health. All identified records were further verified by chart review.

Extensive, standardized reviews of VA's EMR were completed for VA visit encounters and admissions of suspected dengue cases, identified by the above methods in both Puerto Rico and Florida. In Puerto Rico, epidemic years were 2007 and 2010 and in Key West, Florida, epidemic years were 2009–2010 [3], [4], [5], [9]. Cases were grouped into 3 categories: confirmed (positive DENV polymerase chain reaction [PCR], anti-DENV Immunoglobulin M [IgM] seroconversion, ≥4-fold rise in anti-DENV Immunoglobulin G [IgG]); probable (anti-DENV IgM present with a Positivity/Negativity [P/N] ratio ≥2); and suspected (a clinically compatible case, epidemiologically linked to a confirmed or probable case or with travel to a dengue endemic country or presence at a location with an ongoing outbreak within the previous 2 weeks of dengue-like illness, with fever and 2 or more of the following symptoms: retro-orbital or ocular pain, headache, rash, myalgia, arthralgia, leukopenia, or hemorrhagic manifestations without confirmatory laboratory testing or incomplete laboratory testing) [11]. Laboratory testing in Florida was done at hospital or commercial labs and at health departments and in Puerto Rico dengue testing was done at the CDC Division of Vector-Borne Diseases in the Dengue Branch in San Juan, Puerto Rico. In Florida, testing was primarily serologic until 2010 when Florida Department of Health acquired DENV PCR testing capabilities. Patient demographics and clinical signs and symptoms were extracted from encounter notes in the EMR. The extracted EMR data included age, gender, whether a patient was hospitalized and/or received intensive care unit (ICU) care, whether they received platelet or packed red blood cell transfusions, and laboratory results such as dengue laboratory testing, platelet count, hematocrit, and white blood cell count (WBC). Documented symptoms such as fever, arthralgia, myalgias, headache, eye pain, skin manifestations/rash (including petechiae), gastrointestinal (GI) symptoms (nausea, vomiting, and diarrhea), upper respiratory infection (URI) symptoms (cough, nasal congestion, sore throat), and any documentation of bleeding were also extracted from the EMR. Patients noted to have been treated at non-VA hospitals or with other diagnoses or other reasons for their symptoms were excluded.

Clinical and laboratory data from EMRs were reviewed and classified based on the 2010 CSTE dengue case definition [11]. Confirmed and probable cases were combined since they were patients that had at least one positive dengue test. Using Epi Info (CDC, Atlanta, GA), Mantel-Haenszel or Fisher exact tests (if a count was less than 5) were used to compare categorical count data with a p-value≤0.05 signifying a statistical difference. Continuous variables were compared using SAS 9.2 (SAS Institute, Cary, NC) Student's t-test.

## Results

**Figure 1:**
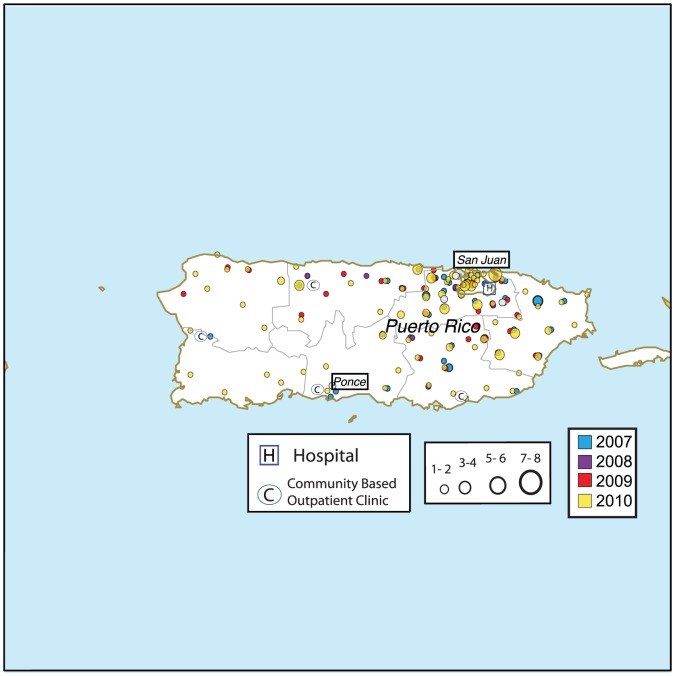
VA dengue cases in Puerto Rico. Location of dengue cases (confirmed/probable/suspected) presenting to Puerto Rico VAs based on zip code of residence, 2007–2010.

**Figure 2:**
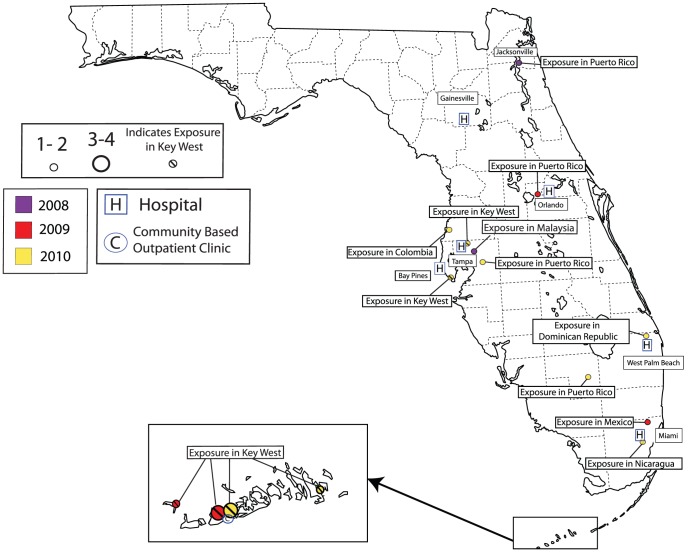
VA dengue cases in Florida. Location of dengue cases (confirmed/probable/suspected) with place of exposure presenting to Florida VAs based on zip code of residence, 2007–2010 (No cases were identified in 2007).

**Figure 3:**
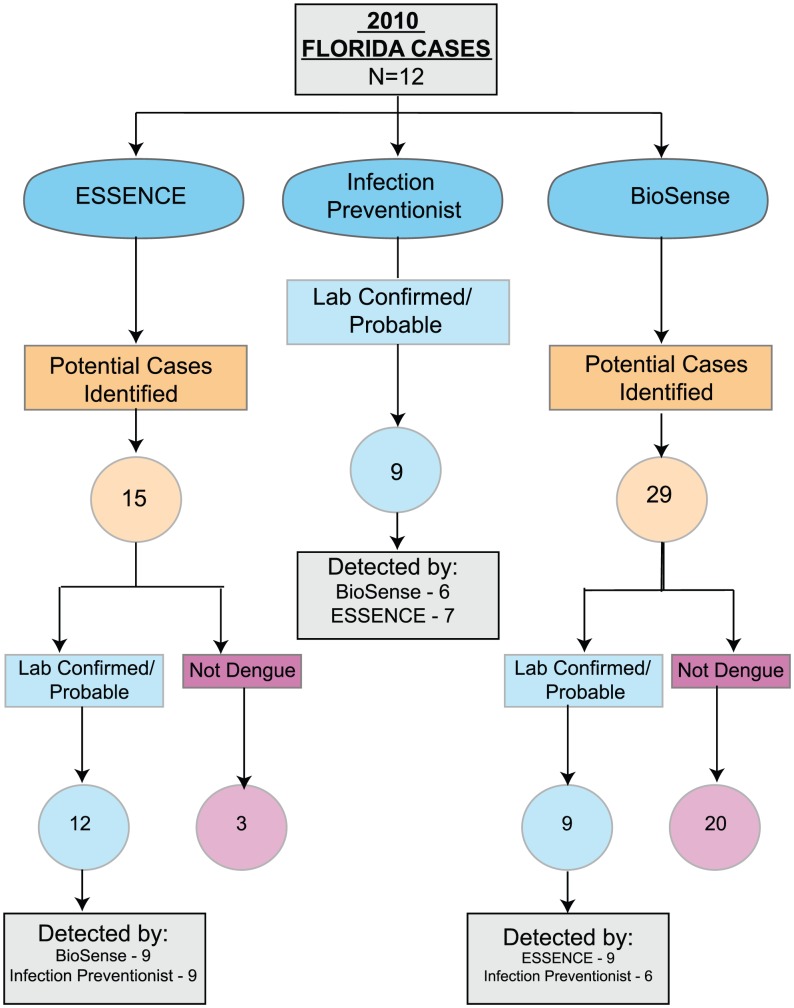
Comparison of electronic and manual surveillance case identification. Flow diagram of identification of 2010 Florida dengue confirmed/probable cases utilizing ESSENCE, infection preventionists, and BioSense.

In VA facilities in the US, including territories as well as the Philippines, there were a total of 339 VA cases of dengue identified between 2007 through 2010. Of those 339 cases, 288 and 21 dengue cases were identified in Puerto Rico and Florida, respectively. The 30 remaining cases were acquired outside the continental US while patients were traveling or in VA facilities located in other US territories in dengue endemic areas (Table 1). All 288 Puerto Rico dengue cases are believed to have been acquired in Puerto Rico. Dengue cases in Puerto Rico presented to VA facilities in San Juan, Ponce, Mayaguez and Arecibo (Figure 1). Twelve of 21 Florida cases were acquired in Key West, Florida, and 9 were acquired while traveling outside Florida in dengue endemic areas from 2007–2010 (Figure 2). In the 2009 analysis, there was only one patient included presenting to a Florida VA who was exposed in Puerto Rico before the start of the Florida epidemic in July 2009. In Puerto Rico, there were 65 cases in 2007, 13 in 2008, 30 in 2009, and 180 in 2010. In Florida, there were 0 cases in 2007, 2 in 2008, 7 in 2009, and 12 in 2010. Dengue surveillance in Florida started in 2010 allowing a comparison between methods of capturing cases. Out of the 12 confirmed/probable cases identified in Florida in 2010, ESSENCE, infection preventionists, and BioSense identified 12 (12/12, 100%), 9 (9/12, 75%), and 9 (9/12, 75%) confirmed/probable cases of dengue respectively (Figure 3). Although not statistically different, there were 5.5 and 9.5 times as many cases during epidemic years in comparison to non-epidemic years during 2007–2010 (245 vs. 43 cases in Puerto Rico and 19 vs. 2 cases in Florida). Overall, VA dengue cases in Florida and Puerto Rico presented at a mean age of 55 years old (range 21 to 90 years) and 94% (289/309) were male. Reported symptoms and signs were fever (298/305, 98%), arthralgias/myalgias (252/276, 91%), eye pain (103/168, 61%), thrombocytopenia [platelets <150 K/mm^3^] (270/305, 89%), headache (203/247, 82%), GI symptoms (181/268, 68%), leukopenia [WBC <4 K/mm^3^] (188/305, 62%), documented skin manifestation/rash (including petechiae) (104/227, 46%), URI symptoms (107/285, 46%), and bleeding (16/224, 7%). Five percent of patients received a platelet transfusion. Dengue diagnostic testing being performed among suspected or confirmed/probable cases was significantly different between facilities in Puerto Rico and Florida, respectively (61% (176/288) and 100% (21/21), p<0.01). In 2010, one patient in Florida had DENV PCR testing performed and was identified as DENV-1, which was the serotype identified by others during this time period in Key West, Florida [10]. In contrast to Florida, Puerto Rico performed more DENV PCR testing (Florida 1/21 cases vs. Puerto Rico 118/288 cases, p<0.01). DENV-1 was the predominant serotype for both epidemic and non-epidemic years in Puerto Rico, accounting for 52/79 (66%) of all serotyped isolates. There was an increase in DENV-4 isolates identified in Puerto Rico during 2010 vs. 2009 (12 vs. 1 case). No patients died as a result of dengue or met criteria for DHF or DSS.

**Table 1:** Patients with dengue diagnosed outside of Puerto Rico and Florida, 2007–2010.

Year	VA Facility	City, State	Seen for Dengue	Place of Exposure
2007	Twin Falls Community-based Outpatient Clinic	Twin Falls, Idaho	Yes, in follow-up	Cook/Tonga Islands
2007	VA Central California Healthcare System	Fresno, California	Yes, active disease	Costa Rica
2007	Southern Nevada Healthcare System	Las Vegas,Nevada	Yes, in follow-up	Philippines
2007	Martinez VA Community-based Outpatient Clinic	Martinez, California	Yes, active disease	Puerto Rico
2007	Southeast Louisiana Veterans Healthcare System VA	New Orleans, Louisiana	Yes, active disease	Honduras
2007	Brooklyn Campus of VA NY Harbor Healthcare System	Brooklyn, New York	Yes, active disease	Guyana
2007	Northport VA Medical Center	Northport, New York	Yes, active disease	Puerto Rico
2007	San Bruno Community-based Outpatient Clinic	San Bruno, California	Yes, in follow-up	Brazil
2007	McAllen Community-based Outpatient Clinic	San Antonio, Texas	Yes, active disease	Mexico
2008	American Samoa Community-based Outpatient Clinic	Pago Pago, American Samoa	Yes, active disease	American Samoa
2008	American Samoa Community-based Outpatient Clinic	Pago Pago, American Samoa	Yes, active disease	American Samoa
2008	American Samoa Community-based Outpatient Clinic	Pago Pago, American Samoa	Yes, active disease	American Samoa
2008	American Samoa Community-based Outpatient Clinic	Pago Pago, American Samoa	Yes, active disease	American Samoa
2008	Bath VA Medical Center	Bath, New York	Yes, active disease	Venezuela
2008	Puget Sound Healthcare System American Lake Division	Seattle, Washington	Yes, active disease	Philippines
2008	Saint Croix Community-based Outpatient Clinic	Kings Hill, Virgin Islands	Yes, in follow-up	St. Croix
2009	White River Junction VA Medical Center	White River Junction, Vermont	Yes, active disease	Haiti
2009	San Jose Outpatient Clinic	San Jose, California	Yes, in follow-up	Ecuador
2009	St Louis VA Division - John Cochran Division	Saint Louis, Missouri	Yes, active disease	Honduras
2010	Manila Outpatient Clinic	Pasay City, Philippines	Yes, active disease	Philippines
2010	James J. Peters VA Medical Center	Bronx, New York	Yes, active disease	Puerto Rico
2010	McCafferty Community-based Outpatient Clinic	Cleveland, Ohio	Yes, active disease	Puerto Rico
2010	North Chicago (Capt. James Lovell) Federal Healthcare Center	North Chicago, Illinois	Yes, active disease	Puerto Rico
2010	East Orange Campus of the VA New Jersey Healthcare System	East Orange, New Jersey	Yes, active disease	Dominican Republic
2010	Gene Taylor Community-based Outpatient Center	Mount Vernon, Missouri	Yes, active disease	Guatemala
2010	Michael DeBakey VA Medical Center	Houston, Texas	Yes, active disease	Unable to determine
2010	Manhattan Campus of VA NY Harbor Healthcare System	New York, New York	Yes, active disease	Puerto Rico
2010	Philadelphia VA Medical Center	Philadelphia, Pennsylvania	Yes, active disease	Puerto Rico
2010	Portland VA Medical Center	Portland, Oregon	Yes, active disease	Venezuela
2010	VA Greater Los Angeles Healthcare System	Los Angeles, California	Yes, active disease	Mexico

Confirmed/probable cases were significantly more likely than suspected cases to be hospitalized (69% vs. 47%, p<0.01), to have thrombocytopenia [platelets <150 K/mm^3^] (96% vs. 84%, p<0.01), and to have leukopenia [WBC <4 K/mm^3^] (80% vs. 51%, p<0.01). Confirmed/probable cases were significantly less likely to report URI symptoms (20% vs. 50%, p<0.01) compared to suspected cases (Table 2). Forty-two percent of suspected patients had incomplete dengue diagnostic testing performed (a single serologic test performed with no convalescent sample submitted).

**Table 2:** Patient characteristics of confirmed/probable versus suspected dengue cases and epidemic cases in Florida and Puerto Rico veterans affairs facilities, 2007–2010.

Patient Characteristics	Confirmed/Probable n = 115	Suspected Cases n = 194	p-value	Epidemic FL † (2009, 2010) n = 19	Epidemic PR † (2007, 2010) n = 245	p-value
Mean Age	54 (Range 23–82)	56 (Range 21–90)	0.47	58 (Range 27–74)	55 (Range 21–90)	0.45
Male	106 [92%]	183 [94%]	0.46	16 [84%]	230 [94%]	0.13
Any Dengue Testing	115 [100%]	82 [42%]	NA	19 [100%]	151 [62%]	**<0.01**
Hospitalization at VA	79 [69%]	92 [47%]	**<0.01**	6 [32%]	137 [56%]	**0.04**
Received ICU Care	6/79 [8%]	5/92 [5%]	0.57	2/6 [33%]	7/137 [5%]	**0.046**
Received Platelet Transfusion	7/115 [6%]	7/194 [4%]	0.31	0/19 [0%]	12/245 [5%]	1
Fever	111/114 [97%]	187/191 [98%]	0.52	16/18 [89%]	238/242 [98%]	0.58
Arthralgias/Myalgias	97/107 [91%]	155/169 [92%]	0.76	14/17 [82%]	205/222 [92%]	0.16
Thrombocytopenia (Platelets <150 K/mm^3^)	109/114 [96%]	161/191 [84%]	**<0.01**	12/16 [75%]	217/245 [89%]	0.12
Headache	82/100 [82%]	121/147 [82%]	0.95	8/15 [53%]	163/194 [84%]	**<0.01**
Any GI Symptom(s)	78/110 [71%]	103/158 [65%]	0.33	8/14 [57%]	152/217 [70%]	0.23
Leukopenia (WBC <4 K/mm^3^)	91/114 [80%]	97/191 [51%]	**<0.01**	10/16 [63%]	150/245 [61%]	0.92
Eye Pain	49/75 [65%]	54/93 [58%]	0.34	3/10 [30%]	88/138 [64%]	**0.05**
Skin Manifestations/Rash (Including Petechaie)	50/96 [52%]	54/131 [41%]	0.11	10/17 [59%]	81/187 [43%]	0.22
Any URI Symptom(s)	19/96 [20%]	69/139 [50%]	**<0.01**	5/15 [33%]	87/191 [46%]	0.36
Bleeding	9/93 [10%]	7/131 [5%]	0.22	1/10 [10%]	12/192 [6%]	0.49

† Includes suspected, probable and confirmed dengue cases.

During the epidemic years (2009–2010) in Florida, 12 patients were linked to travel to or residence in Key West, Florida, while 7 patients had documented travel outside of Florida to other endemic areas prior to their diagnosis of dengue (Figure 2). The 12 patients with exposure in Key West presented to VA facilities in Key West (8), Miami (2), Tampa (1) and Bay Pines (1) (Figure 2). These groups were compared and no statistical difference was identified for the patient characteristics listed in Table 2; however, there was a trend towards more hospitalizations and more ICU care received by patients that had traveled outside of the continental US (data not shown). The 7 patients with documented travel outside of Florida were included as part of the Florida epidemic analysis since they presented to a Florida VA facility during the evaluation period.

Florida and Puerto Rico epidemic cases (suspected and confirmed/probable) were compared to each other (Table 2). In Florida, cases were significantly more likely to have any dengue diagnostic testing completed (100% vs. 62%, p<0.01), less likely to be hospitalized (32% vs. 56%, p = 0.04) but more likely to receive ICU care (33% vs. 5%, p = 0.05). In Florida, patients were significantly less likely to report symptoms of headache (53% vs. 84%, p = 0.01) and eye pain (30% vs. 64%, p = 0.05). The rest of the patient characteristics were not significantly different. Although the numbers are small, the Florida and Puerto Rico non-epidemic years were compared and there were no significant differences in patient characteristics between these groups (data not shown).

Epidemic years of dengue in Puerto Rico and Florida were combined and compared to non-epidemic years. None of the patient characteristics were significantly different between the groups. However, there was a higher proportion of hospitalizations and documented skin manifestations/rash in the non-epidemic years (data not shown).

## Discussion

The re-introduction of DENV in the continental US has made it an important infection for VA providers to understand, especially since approximately two-thirds of confirmed/probable cases and almost half of the suspected cases were hospitalized. The majority of endemic VA dengue cases during 2007–2010 were identified in Puerto Rico and Florida. In VA medical facilities in Florida, locally acquired dengue was limited to Key West, Florida, while all other VA cases detected in Florida during the epidemic had an exposure outside of the state. While the number of VA dengue cases in Florida decreased in 2011 to 3 imported cases and is no longer showing sustained local transmission, the potential for further spread of DENV infection in Florida and other parts of the US is possible. Therefore, it is important to understand the clinical presentation, diagnostic testing patterns and epidemiology of dengue within the VA system.

Overall, our dengue cases presented with expected signs and symptoms of fever, arthralgias/myalgias, headache, skin manifestations/rashes, headache, thrombocytopenia, and leukopenia. Clinically, our Florida cases appeared to be no different than those found in Puerto Rico. The similarity of clinical symptoms was not surprising since a DENV-1 strain, related to other Central American strains of dengue, was the predominant serotype identified in the Florida epidemic [8]. Based on the 2010 CSTE dengue case definition, confirmed/probable patients had similar characteristics as suspected cases. However, as expected based on the definition of a confirmed/probable case, all of these patients had dengue diagnostic testing, while only 42% had testing in the suspected group (no lab test was needed to meet this definition and some suspected patients had initial dengue diagnostic testing performed but did not have convalescent serologic testing to confirm a diagnosis). Of note, less than two-thirds of suspected dengue cases in Puerto Rico received any dengue diagnostic testing, even though free diagnostic testing is available in Puerto Rico through CDC. In some cases, testing was never ordered and in others, samples were rejected because the CDC-required paperwork was either incomplete or not filled out properly. This finding highlights the need for additional education among VA providers regarding availability of testing and collection of required patient information. We also found that hospitalization, thrombocytopenia, and leukopenia rates were higher in the confirmed/probable group. Interestingly, URI symptoms were less likely in confirmed/probable cases; implying patients with URI symptoms were less likely to have dengue [15]. However, 20% of confirmed/probable cases reported URI symptom(s) so their presence does not preclude the diagnosis of dengue and testing patients that have URI symptoms and are suspected of having dengue remains important.

The cases identified in Florida during the Key West epidemic were similar in nature and severity to those seen during epidemic years in Puerto Rico except for the significantly increased amount of testing and increase in cases receiving ICU care in Florida. Interestingly, there was a lower incidence of overall hospitalization for dengue in Florida during the epidemic years. This suggests that even though fewer patients were hospitalized in Florida, when they were hospitalized they received ICU level care. However, although the numbers are small, the number of hospitalized cases in Florida was low and when chart reviews were performed on the patients receiving ICU level care it revealed either atypical presentations or a provider's decision for closer monitoring in an ICU setting, suggesting a relative discomfort in treating dengue in Florida facilities compared to Puerto Rico. There were a lower percentage of cases presenting with headache and eye pain in Florida, however, this may be related to fewer providers asking patients and documenting these symptoms in the EMR. In addition, the cases of dengue during epidemic and non-epidemic years had similar characteristics.

Limitations of our study included a small cohort number that was predominantly older males. In addition, some Veteran cases could have been missed if they were treated at outside facilities and not reported or if they were not coded as having dengue. Dengue PCR was not as commonly used in the Florida cases so we are unable to determine the most common serotype involved with the VA dengue cases in the continental US, or to have samples to do further molecular typing and sequencing. Since we combined confirmed and probable dengue cases it is possible that we slightly overestimated the number of actual dengue cases since elevated anti-DENV IgM may be due to a cross reactivity with other flaviviruses (West Nile virus, St. Louis encephalitis virus, Japanese encephalitis virus, and yellow fever virus), although this is unlikely as there are few places where these viruses co-circulate or where these conditions cannot be differentiated clinically [16], [17]. In addition, many Veterans are vaccinated against yellow fever which can have cross reactivity with dengue serologic testing [16]. However, the number of probable cases was small therefore it is unlikely to have greatly affected our analysis. Unfortunately, primary/secondary dengue infection status is not reliably documented in our EMR making it impossible to compare prior dengue exposure to symptom severity.

The primary goal of our study was not to compare different surveillance system performance for DENV detection. However, accurate DENV case finding required the combination of two electronic biosurveillance systems (ESSENCE and BioSense), as well as infection preventionist manual surveillance efforts at VA facilities. These electronic biosurveillance systems currently rely on outpatient diagnostic encounter codes, ICD-9, which can be searched by syndrome or individual codes. ICD-9 coding for outpatient visits in VA may not be completely accurate, and likely underestimates the true number of cases, particularly in those cases where confirmatory laboratory testing was not obtained, or was obtained and results were not available at the time of encounter close-out. Syndromic surveillance includes additional, non-specific ICD-9 codes (i.e., fever and rash), which can further reduce the specificity of the diagnosis. In addition, DENV or syndrome ICD-9 codes could reflect prior outpatient encounters for DENV disease, and contribute to an overestimation of the number of cases. Infection preventionists can access multiple data domains in the EMR (including history, laboratory data, and treatment), which help refine whether a potential DENV case is likely to be a confirmed or probable case. In addition, infection preventionists can help facilitate obtaining convalescent blood samples to further help confirm diagnoses. As demonstrated in Figure 3 no system of identifying cases was perfect, both infection preventionists and BioSense were able to identify 9 out of the 12 confirmed/probable cases. ESSENCE was able to capture all 12 cases, however, misidentified 3 cases. Because of the reduced specificity of electronic biosurveillance systems, VA is enhancing VA's ESSENCE system by including vital signs (temperature), laboratory orders and results, inpatient admission data, outpatient encounter severity codes, telephone care encounter data, and pharmacy prescription data, in addition to ICD-9 encounter codes, which will improve specificity and automate much of what infection preventionists currently must review by hand. Until our enhanced system is available, utilization of an electronic surveillance system in addition to manual surveillance by infection preventionists will remain important.

Although indigenous cases of DENV infection are rare in the continental US, after the epidemic of dengue in Key West, Florida, greater attention was placed on dengue surveillance, education and public health reporting. The VA Office of Public Health, CDC and Florida Department of Health collaborated on providing educational materials including a VA Dengue Health Alert that was produced in July 2010 to help educate VA providers on the presence of dengue in Florida. The alert advised providers to be vigilant for symptoms of dengue, to report suspected cases to local and state health departments, and to obtain appropriate laboratory testing for confirmation. Laboratory testing will hopefully become more widely available now that a DENV reverse transcription polymerase chain reaction (RT-PCR) assay developed by the CDC has been approved by the US Food and Drug Administration [18]. Additional details on laboratory testing algorithms and clinical guidance are available on the CDC website [19]. Increased efforts are necessary to improve dengue awareness in VA through patient and clinician education, and which emphasizes the need for testing, accurate coding of potential dengue cases and appropriate reporting to county and state health officials.
